# Geographical distribution of radon and associated health risks in drinking water samples collected from the Mulazai area of Peshawar, Pakistan

**DOI:** 10.1038/s41598-024-55017-5

**Published:** 2024-03-13

**Authors:** Syed Samran Ali Shah, Abdul Rahim Asif, Manzoor Ilahi, Haseeb Haroon, Ihtisham Islam, Adnan Qadir, Irfan Nisar, Malik Muhammad Usman Sani, Rashid Iqbal, Muhammed Habib ur Rahman, Muhammad Arslan, Mona S. Alwahibi, Mohamed S. Elshikh, Allah Ditta

**Affiliations:** 1 School of Computing and Engineering, University of West London, Ealing, London, UK; 2 National Centre of Excellence in Geology, University of Peshawar, Peshawar, 25120 Pakistan; 3 GIS & Space Applications in Geosciences (G-SAG) Lab, National Centre of Excellence in Geology, University of Peshawar, Peshawar, 25120 Pakistan; 4 Department of Geology, Shaheed Benazir Bhutto University Sheringal, Dir (U), 18000 Pakistan; 5 Pakistan Museum of Natural History, Shakarparian National Park, Garden Ave, Islamabad, 44000 Pakistan; 6 Department of Agronomy, The Islamia University of Bahawalpur, Bahawalpur, 63100 Pakistan; 7 Agroecology and Organic Farming Group, Institute of Crop Science and Resource Conservation, University of Bonn, Bonn, Germany; 8 Crop Science, INRES, University of Bonn, Germany, Bonn, Germany; 9 Department of Botany and Microbiology, College of Science, King Saud University, 11451 Riyadh, Saudi Arabia; 10 Department of Environmental Sciences, Shaheed Benazir Bhutto University Sheringal, Dir (U), 18000 Pakistan; 11 School of Biological Sciences, The University of Western Australia, 35 Stirling Highway, Perth, WA 6009 Australia

**Keywords:** Radon, Inhalation, Ingestion, Spatial distribution, Health risk assessment, Environmental sciences, Risk factors

## Abstract

Geospatial methods, such as GIS and remote sensing, map radon levels, pinpoint high-risk areas and connect geological traits to radon presence. These findings direct health planning, focusing tests, mitigation, and policies where radon levels are high. Overall, geospatial analyses offer vital insights, shaping interventions and policies to reduce health risks from radon exposure. There is a formidable threat to human well-being posed by the naturally occurring carcinogenic radon (^222^Rn) gas due to high solubility in water. Under the current scenario, it is crucial to assess the extent of ^222^Rn pollution in our drinking water sources across various regions and thoroughly investigate the potential health hazards it poses. In this regard, the present study was conducted to investigate the concentration of ^222^Rn in groundwater samples collected from handpumps and wells and to estimate health risks associated with the consumption of ^222^Rn-contaminated water. For this purpose, groundwater samples (n = 30) were collected from handpumps, and wells located in the Mulazai area, District Peshawar. The RAD7 radon detector was used as per international standards to assess the concentration of ^222^Rn in the collected water samples. The results unveiled that the levels of ^222^Rn in the collected samples exceeded the acceptable thresholds set by the US Environmental Protection Agency (US-EPA) of 11.1 Bq L^−1^. Nevertheless, it was determined that the average annual dose was below the recommended limit of 0.1 mSv per year, as advised by both the European Union Council and the World Health Organization. In order to avoid the harmful effects of such excessive ^222^Rn concentrations on human health, proper ventilation and storage of water in storage reservoirs for a long time before use is recommended to lower the ^222^Rn concentration.

## Introduction

In the Earth's topmost layer, rocks and soils contain minuscule amounts of radioactive elements that undergo natural decay and produce an odorless and tasteless gas i.e. Radon^1,2^. The radioactive gas Radon is chemically inert, emits α-particles, and exists in three isotopes naturally i.e. Radon (^222^Rn), Actinon (^219^Rn), and Thoron (^220^Rn). The most stable isotope, ^222^Rn, is created when Uranium (^238^U), which makes up 99.3% of all the uranium in the earth's crust decays. Similarly, Actinon (^219^Rn) is produced through the decomposition of Uranium (^235^U), whereas, Thoron (^220^Rn) is produced via the decomposition of Thorium (^232^Th). The stable and common radon isotope is ^222^Rn, which has a half-life of 3.8 days^3,4^. After decaying, it releases 5.49 meV α-particles that form radioactive isotopes in all terrestrial water sources, including lakes, rivers, seas, wells, and even atmospheric precipitation^5–7^.

The ^222^Rn is readily soluble in water, and environmental conditions like pressure and temperature greatly affect its solubility^8,9^. If ^222^Rn is found in the empty spaces of soil and rocks, it will enter the water^10^. The quantity of ^222^Rn in the underground water is initiated by the existing environmental conditions. When using water for domestic purposes, it is most probable to be exposed to ^222^Rn^11,12^. There are two potential routes through which individuals can come into contact with ^222^Rn in water: either by consuming water that has been contaminated or by inhaling the ^222^Rn that is generated from domestic water sources. These routes can lead to possible health issues like stomach and lung cancer in humans^13^.

In many parts of the world, ^222^Rn, a common carcinogenic gas, poses serious health issues to humans^14^. Humans can breathe in or ingest radon gas when it is mixed in drinking water or other household functions^15,16^. Similarly, exposure to ^222^Rn through a variety of sources could pose substantial health concerns to human populations^17–19^. According to a report released by USEPA in 1999, the inhalation of radon gas is accountable for a staggering 89% of fatalities caused by lung cancer and 11% of cases of ingestion-related stomach cancer. Chromosome variations are more likely to occur in workers with very high ^222^Rn exposure^20^. The greatest rates of gastrointestinal and respiratory effects from malignant tumors in China were due to ^222^Rn exposure^21^. Lately, ^222^Rn concentrations and related health dangers have received attention in many countries throughout the world e.g. Austria^22^, Poland^23^, India^24,25^, Malaysia^26,27^, Nigeria^28^, etc.

Health risks associated with ^222^Rn contamination have received great attention in the developed nations of the world. Public awareness of ^222^Rn has been raised through a variety of network media, including television and newspapers. Reference and action values for radon have been determined. Various researchers in different conferences and workshops have identified ^222^Rn in air, and water and proposed various techniques to tackle this issue. New radon-free techniques are already being used and introduced. There are now dedicated ^222^Rn measurement labs that are well-equipped. In other nations, ^222^Rn oversight and control are even now mandated by law.

Both the campaign through commercials and the ^222^Rn measuring programs have not yet been launched on a national level in developing nations like Pakistan. The degree of ^222^Rn concentration in a few particular regions and organizations has only been examined through very small-scale personal efforts for academic interest^29–32^. To comprehensively examine the extent of ^222^Rn contamination in water sources and assess its potential health implications, it is imperative to conduct rigorous investigations across various disciplines. Based on this hypothesis, the present study was conducted to assess the spatial distribution patterns of radon in drinking water sources obtained from different depths, and subsequently determine the associated health risks across different age groups in the Mulazai area. Additionally, the study aimed to compare the average annual effective dosage of ^222^Rn in drinking water with the globally recommended maximum limit.

The decision to delve into this research area stemmed from the pressing concern raised by individuals who brought water samples to our research institute for testing. Their claim regarding the higher incidence of cancer in the Mulazai area compared to Peshawar sparked our interest and raised alarms about potential water-related health risks. Investigating this discrepancy became crucial to understanding the possible link between water quality and the elevated cancer rates in the Mulazai region.

## Materials and methods

### Geography

Mulazai town is situated in the western region of the Peshawar basin. Peshawar basin alone occupies an area of around 8000 km^2^ and is located in the northwest of Pakistan^33^. Apart from its southeastern side, which allows water to flow out to the Indus River, the region is encompassed by rugged and mountainous terrain on all other sides. The Attock-Cherat range in the south is characterized by sedimentary rocks of Paleogene and Neogene, while the northern border is defined by the Himalayan ranges which comprise Precambrian and Tertiary rocks^34^. The Indus River is the recipient of three primary rivers that flow towards the east from the valley. These include the Kabul River, which enters from the northwest, as well as the Kalpani, and Swat Rivers, both of which originate from the northern highlands.

### Geological characteristics

**Figure 1:**
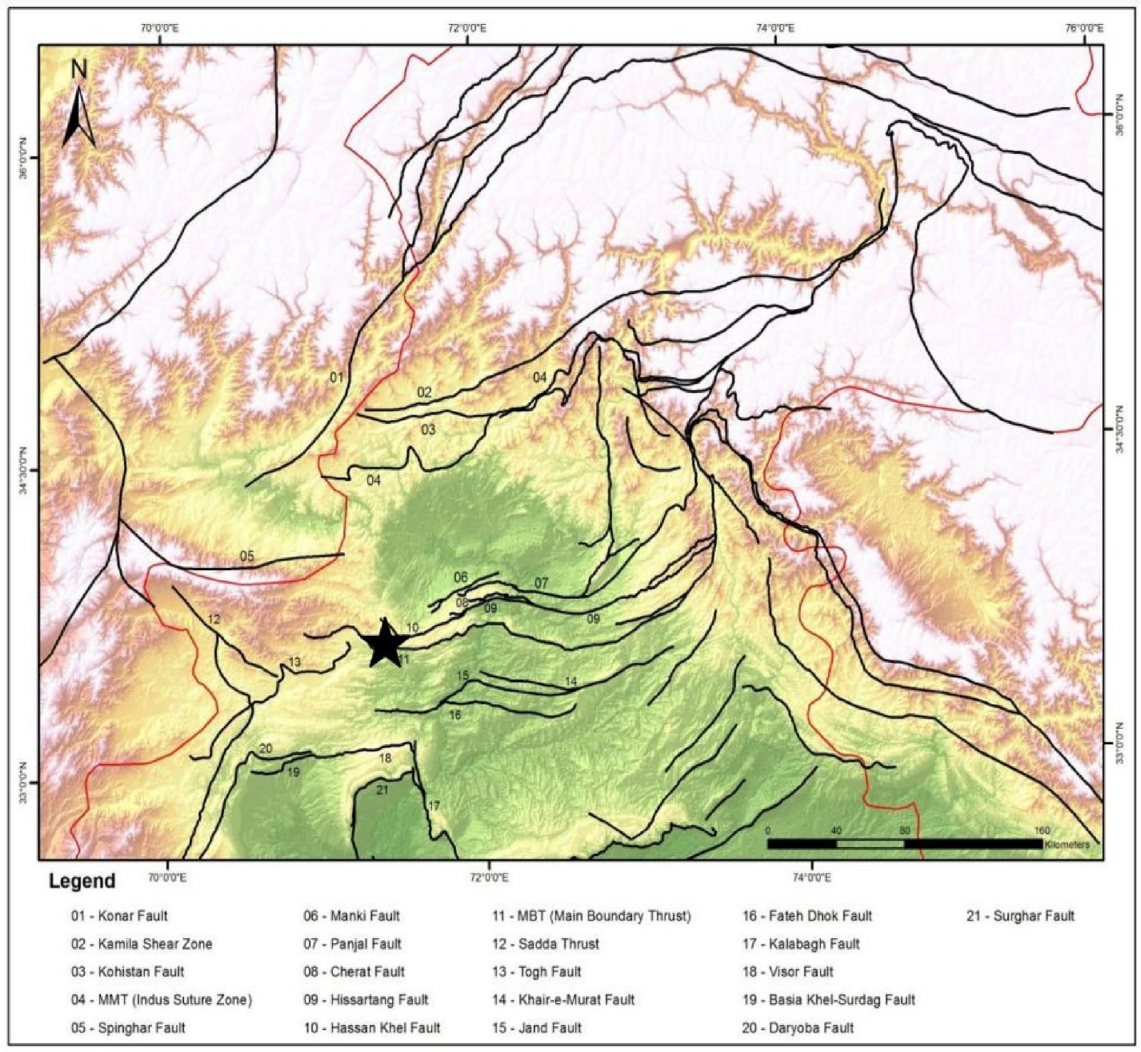
Map showing Regional Tectonics and Geology of the study area.

Peshawar Basin contains a significant amount of Quaternary alluvial sediments, measuring several hundred meters in thickness^33^. Around 2.8 million years ago, sediment deposition commenced in the Peshawar Basin. During the late Miocene to Pliocene, antecedent molasse sediments underwent a phase of folding and thrusting. Consequently, the basin has become saturated with sediments, which have accumulated to a remarkable thickness of up to 300 m within the region^35^. Bordering the basin, we find Quaternary fanglomerates, while the central area primarily consists of micaceous sand, lacustrine deposits, and gravel. These geological formations were shaped by the movement of rivers and other water bodies^36^. The most recent sedimentary rocks found in the Peshawar basin are the Murree Formation rocks, which have undergone extensive oxidation. These rocks can be seen at the edges of the basin^37^. Around 0.6 million years ago, the Attock-Cherat Range experienced a fast uplift that ended the extensive sedimentation within the inter-montane basins as shown in Fig. 1.

### Hydrography

The water table depth in the basin fluctuates between 5 and 40 m^33^. The primary sources of water for household and irrigation uses are bored/drilled wells and dug wells. Bored wells can reach depths of 50–150 m, while dug wells are generally shallower and do not exceed 20 m in depth^34^. The movement of groundwater within the Peshawar Basin typically moves towards the center of the basin and then discharges in an eastern direction towards the Indus Valley. This discharge may occur either as groundwater or as surface water, following infiltration into the river^33^.

The Peshawar region is situated in the northern part of Khyber Pakhtunkhwa and runs alongside the Kabul River. Despite covering only 10% of the total area of the province, it serves as the residence for nearly half of its populace. Situated in the western region of Peshawar, the Khyber Pass is a renowned landmark that offers a convenient route between the Indian subcontinent and Afghanistan. The weather in Peshawar is warm and temperate, with significant precipitation all year round, even during the driest months. The average temperature hovers around 22.3 °C or 72.1 °F, and the yearly precipitation in Peshawar varies from 817 mm to 32.2 inches.

**Figure 2:**
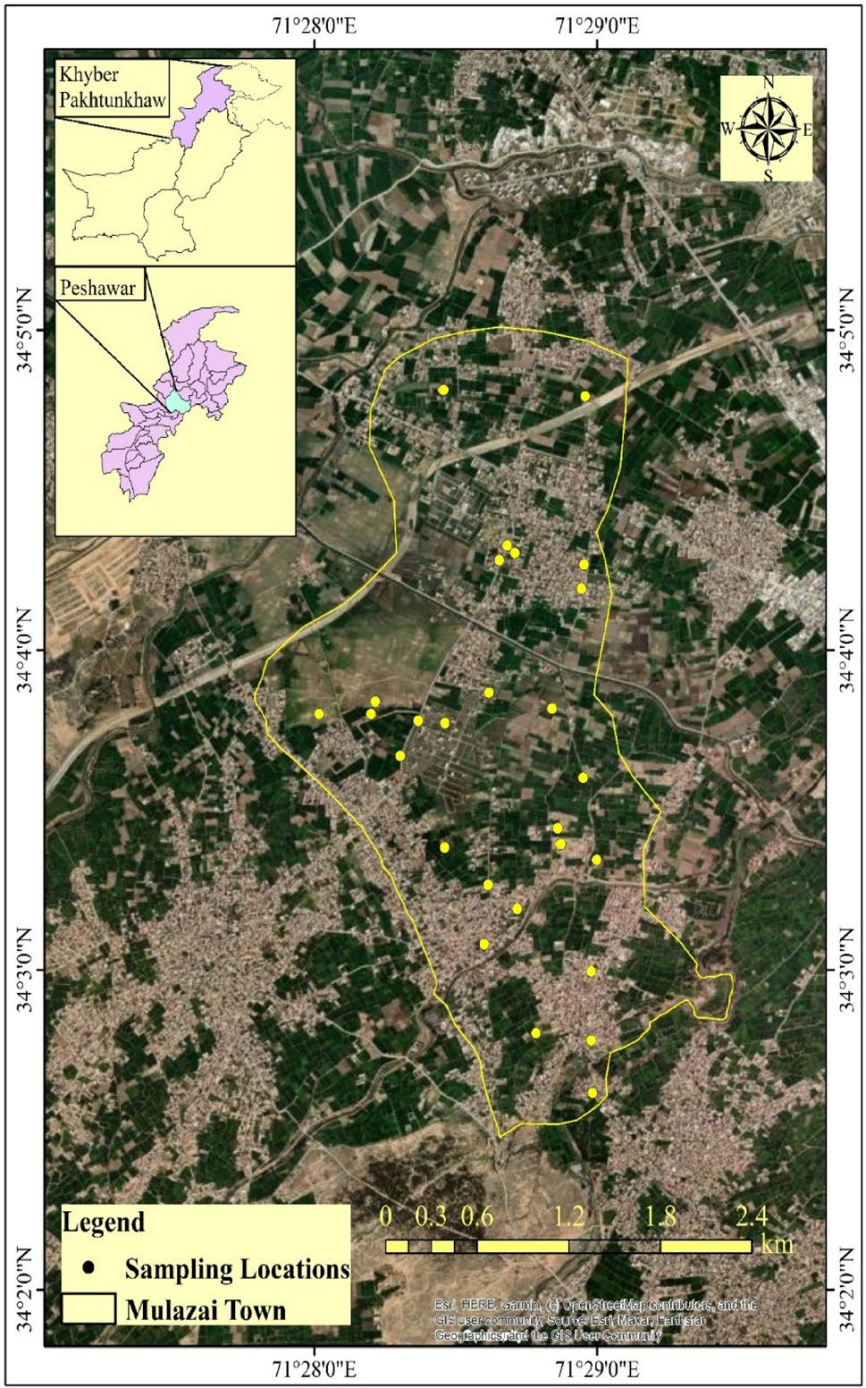
Map showing the sampling area of Mulazi, Peshawar (ArcGIS version 10.7.1, ESRI, Redlands, CA, USA).

Located approximately 16 km northwest of Peshawar city in Pakistan's Khyber-Pakhtunkhwa Province, Mulazai is situated in the western part of the Peshawar Basin, occupying a strategic location along the prominent historic Grand Trunk Road (G.T Road). This road serves as a vital link between the provincial capital of Peshawar and the city of Jalalabad in Afghanistan, extending westward. Geographically, Mulazai is positioned at latitude N 34, 000, 11.2600 and longitude E 71, 250, 00.3900 (Fig. 2).

### Sampling methodology

In mid-January 2016, a comprehensive water sampling campaign was conducted to collect samples for laboratory analysis. The water samples, crucial for assessing the quality and composition of the local water sources, were gathered using industry-standard procedures. To ensure the integrity and reliability of the collected samples, an official vehicle was employed for their transportation to the laboratory. This mode of transport adhered to established protocols, guaranteeing that the samples reached the laboratory in their original state, free from external contamination or alterations. The utilization of an official vehicle underscored the commitment to precision and accuracy in the analytical process, laying the foundation for reliable and meaningful results in subsequent water quality assessments. Thirty water samples were diligently collected from various locations, employing a random selection approach with intervals of 200 m. Meticulous efforts were made to ensure the accuracy of the water sampling process in the study area and its surrounding regions, as illustrated in Fig. 2.

The precise GPS coordinates of each sampling site were meticulously recorded using a Garmin etrex10 device. To guarantee the integrity of the collected samples, a specially designed sampling container was utilized to extract water from taps, boreholes, and wells. Before collection, thorough flushing of the water source was conducted for 5–10 min to minimize the risk of contamination. In preparation for precise ^222^Rn measurement, the glass bottles with a capacity of 250 mL were meticulously cleaned, with particular emphasis on suitability for ^222^Rn assessment. Great care was taken to ensure the proper sealing of the bottles, leaving no room for the entrapment of air during the measurement process. The quantification of ^222^Rn concentration within the collected samples was carried out using a state-of-the-art RAD7 machine, manufactured by the esteemed Durridge Company in the United States. The RAD7 is a Sniffer that detects the 3-min alpha decay of a radon daughter without interference from other radiations. With a 10% standard deviation, the RAD7 Continuous Radon Monitor can measure the EPA action level of 4 pCi/L in less than two hours. The detector composes a comprehensive report at the end of each test. This advanced device facilitated accurate and reliable measurements, further enhancing the overall quality of the study.

### Analysis

An on-site examination was carried out to measure the level of ^222^Rn in the samples. The H_2_O method was used to calculate the ^222^Rn value in water using RAD7 (Durridge Company, USA). Due to the fast rate of decay and bottle leakage of ^222^Rn, the concentration in samples was checked immediately. The conversion coefficients and standard protocols used by the RAD7 H_2_O method are predetermined. The Auto Wat 250 technique was used to analyze water, and the process took 30 min to complete^38^. Following 30 min, the RAD7 device displayed a concentration of ^222^Rn^10,39^. The RAD7 device exhibited exceptional precision in measuring ^222^Rn levels within the 4–400 Becquerels per liter (Bq L^−1^) range, with a moisture level inside the device remaining below 9%^40^. To ensure the reliability and accuracy of the measurements, verified radium samples with 4, 40, and 100 (Bq L^−1^) were employed to generate a standard curve and its subsequent use for the calculation of ^222^Rn concertation in the collected samples. The RAD7 manufacturer's calibration methods were applied to examine these samples for quality control purposes. RAD7 is calibrated by the manufacturer against a master instrument, which, in turn, is calibrated against a standard maintained by the British National Radiological Protection Board (NRPB), known as HPA (Health Protection Agency) since 2004. The overall calibration accuracy is estimated to be about ± 5%^40^.

### Evaluation of annual effective dose

There are two ways that people may experience health problems due to their exposure to ^222^Rn in water. These pathways involve inhalation and ingestion. Inhalation takes place when individuals inhale air containing ^222^Rn, which enters their lungs. Conversely, ingestion occurs when ^222^Rn enters the stomach through the consumption of water contaminated with ^222^Rn. To ascertain the annual average effective doses of ^222^Rn intake (EwIng) and inhalation (EwInh) resulting from water consumption, the equation utilized is the one presented by the United Nations Scientific Committee on the Effects of Atomic Radiation (UNSCEAR) in their 2000 report^41^.

1 $${\text{EWIng}}={{\text{C}}}_{{\text{RnW}}}\times {{\text{C}}}_{{\text{W}}}\times {\text{EDC}}$$

To determine the mean effective dose of ^222^Rn ingestion, expressed in mSv year^−1^ or μSv year^−1^ shown in Eq. (1), it is necessary to employ a formula that takes into account two key factors: CRnW and CW. The former denotes the quantity of ^222^Rn present in water, measured in Bq L^−1^, while the latter represents a weighted approximation of water usage, measured in 3.5 nSv Bq^−1^. The calculation of the effective dose of ingestion relies on the effective dose coefficient (EDC). Additionally, it is crucial to consider the daily water intake for different groups such as adults, children, and infants, which are respectively 0.6, 0.8, and 1.3 L, as this information is vital for determining EwIng^29,42^. An EwInh (mSv year^−1^ or μSv year^−1^) was calculated as adapted by UNSCEAR ^41^: shown in Eq. (2)

2 $${\text{EWInh}}={{\text{C}}}_{{\text{Rn}}}\times {\text{F}}\times {\text{O}}\times {\text{R}}\times {\text{DCF}}$$

The dose derived from exposure to ^222^Rn can be computed utilizing the following exceptional formula: Dose = CRn × F × O × (1/10^4^) × DCF. The symbol CRn represents the concentration of ^222^Rn measured in units of Becquerels per liter (Bq L^−1^). An equilibrium factor denoted as F, with a value of 0.4, signifies the equilibrium between ^222^Rn and its decay products. The symbol O represents the average annual duration an individual spends indoors, which amounts to 7000 h per year. The ratio of ^222^Rn concentration in air to its concentration in water is 10^−4^. Lastly, the conversion factor denoted by DCF, which equals 9 nanosieverts per Becquerel per hour per cubic meter (nSv/(Bq h m^−3^)), is used to compute the radiation dose resulting from ^222^Rn exposure^42,43^.

To ascertain the total yearly effective dose of ^222^Rn, utilize the subsequent formula:

3 $${\text{EWTotal}}={\text{EWIng}}+{\text{EWInh}}$$

EwTotal represents the collective effective dose of ^222^Rn as shown in Eq. (3).

By modifying the equation of ICRP ^44^, the ELCR of ^222^Rn was computed as shown in Eq. (4).

4 $${\text{ELCR}}={\text{H}}\times {\text{DL}}\times {\text{RF}}$$

The calculation for assessing the perilousness of cancer resulting in fatality per unit of radiation exposure (Sievert) relies on three key elements: the mean effective dose (H), the standard lifespan (DL) of 70 years, and the probability of deadly cancer (RF) at 5.5 × 10^−2^ per Sv.

### Geospatial analysis

The rates at which infants, children, and adults consume and breathe in ^222^Rn were computed and demonstrated using the advanced Sigma Plot software version 12.5, developed by Systate Inc. To examine the concentrations of ^222^Rn and EwTotal within a particular age group, a sophisticated technique called interpolation, employing ArcGIS software with inverse distance weighting, was employed. For any geographic point data, such as water quality parameters and chemical concentrations, an interpolation approach is utilized for predicting unknown values using known or collected data.

## Results and discussion

The US EPA ^45^ has not yet established any specific guidelines for the acceptable level of ^222^Rn in drinking water. However, the EPA has made a recommendation for controlling ^222^Rn contamination in public water supplies, proposing a maximum allowable/contaminant limit (MCL) of 11.1 Bq L^−1^. This proposal takes into account the estimated risks associated with inhaling ^222^Rn and assumes a transfer ratio of ^222^Rn from water to air in households, which is approximately 104–1.

### Analysis

**Figure 3:**
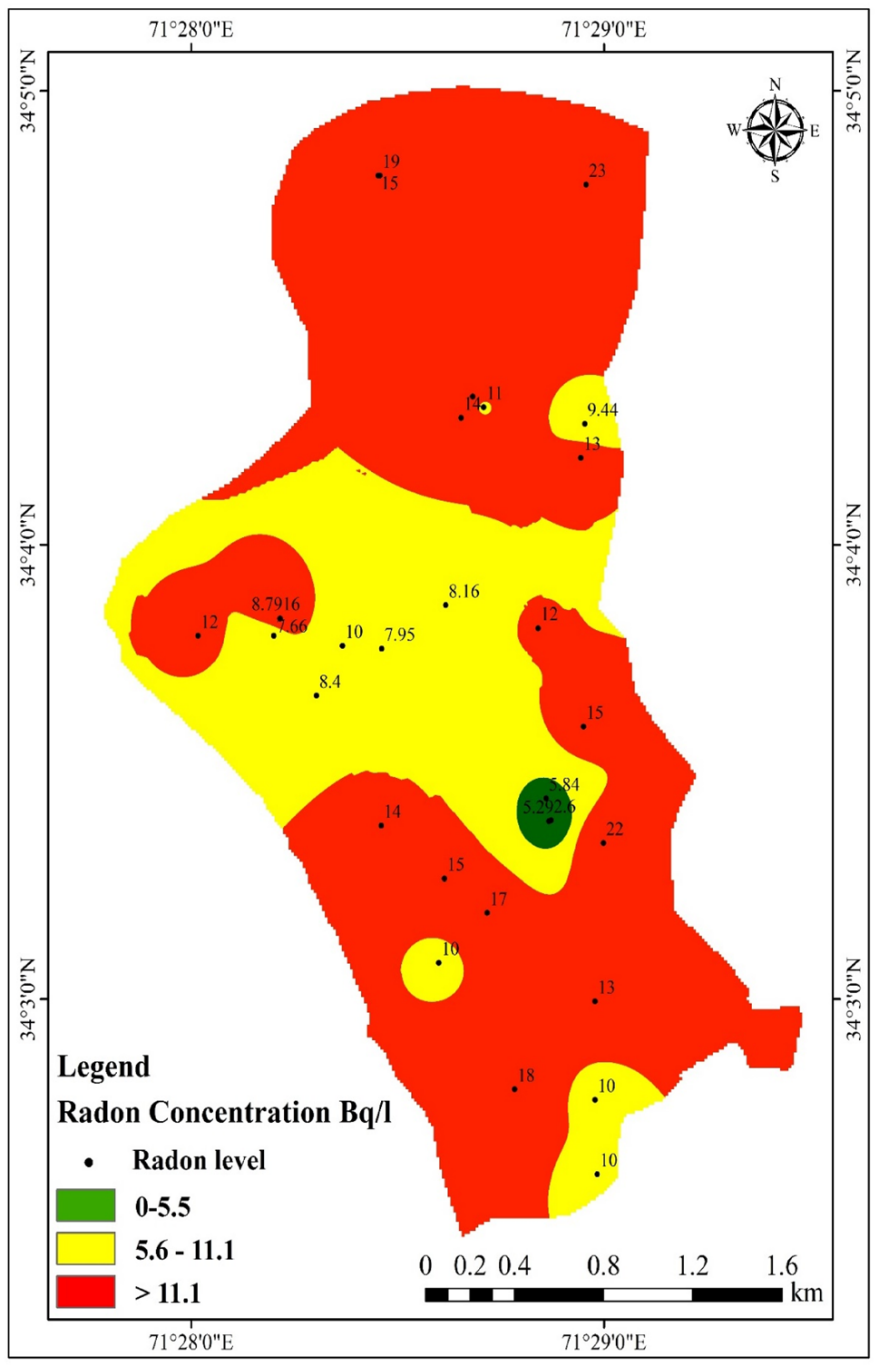
Map showing the concentration of Radon in drinking water samples.

In this study, a total of 30 drinking water samples were collected from the research area to assess the concentration of ^222^Rn. Consequently, the average yearly effective doses to the stomach, lungs, and entire body were calculated for an adult. The analysis revealed that 16 of the samples collected from hand pumps exceeded the MCL of 11.1 Bq L^−1^. Figure 3 illustrates the data, showing that more than half (53.33%) of the water samples obtained from hand pumps had ^222^Rn levels surpassing the EPA's proposed MCL of 11.1 Bq L^−1^. The 30 water samples collected for drinking purposes exhibited a range of ^222^Rn values, with the lowest, highest, and average values being 2.6, 23.0, and 12.0 Bq L^−1^, respectively. The highest recorded ^222^Rn value of 23 Bq L^−1^ was found in a sample collected from a hand pump located at Mulazai Chowk alongside the roadside.

The concentration of ^222^Rn in the drinking water samples of the study area under investigation surpassed the levels found in water samples collected from different regions worldwide by various researchers. For instance, the concentration in the study area exceeded that of Peshawar^43^, Agbabu, Nigeria^28^, Makkah, Saudi Arabia^46^, Penang, Malaysia^27^, Mirpur^31^, and Rajasthan.^12^ On the other hand, it was lower than the concentrations found in Bannu^32^, Gilgit^9^, and Nigeria^47^. A comparison of the ^222^Rn concentration in the study area's drinking water with data from previous studies conducted in various regions and countries is presented in Table 1.

**Table 1:** Comparison of the ^222^Rn concentration in the drinking water of the study area with data from prior studies conducted in various regions and countries.

Location	Country	^222^Rn concentration (Bq L^−1^)	References
Gilgit	Pakistan	275	^9^
Mirpur, AJK	Pakistan	9.46	^31^
Penang	Malaysia	0.06	^27^
Nigeria	Nigeria	1.6–271	^47^
Rajasthan	India	0.62	^12^
Agbabu	Nigeria	30.2 ± 6.7	^28^
Makkah	Saudi Arabia	0.5	^46^
Bannu	Pakistan	10.1–53.1	^32^
Iraq	Iraq	0.12	^48^
Mirpur, AJK	Pakistan	11.1	^49^
Dhaka	Bangladesh	7.13	^50^
Rajasthan	India	4.42	^51^
Kirkuk	Iraq	0.33 ± 0.58	^52^
Karak	Pakistan	9.37 ± 0.4	^53^
Peshawar	Pakistan	8.80 ± 0.80	^43^
EPA	US EPA	11.1	^45^
Peshawar	Pakistan	12.23	Current study

### Causes and sources

The differences in ^222^Rn levels observed in drinking water across these regions can be attributed to a variety of factors, both local and regional. These factors may include the geological composition of the bedrock, mineral composition, soil texture, as well as variations in climate^54,55^.

**Figure 4:**
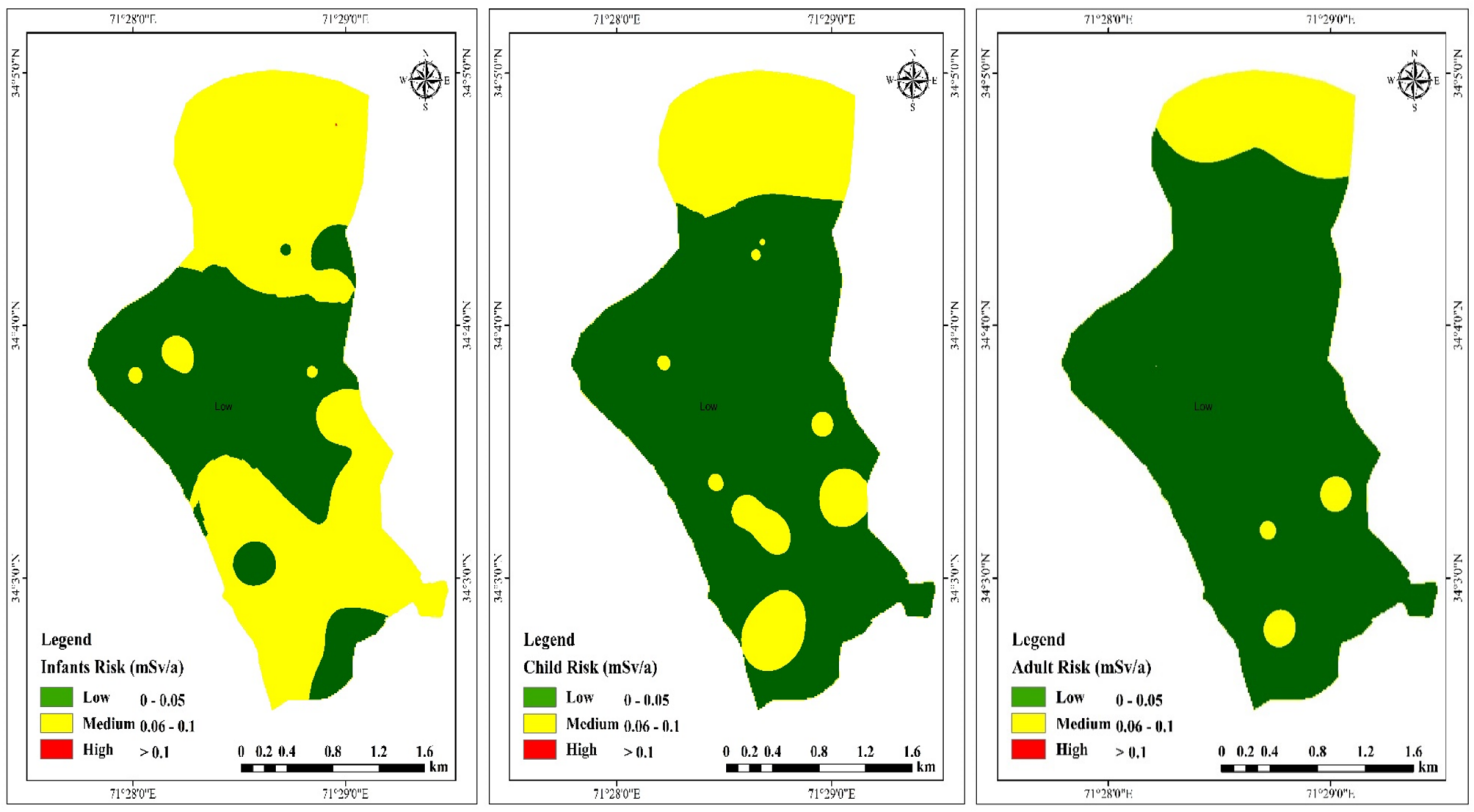
Geospatial map of exposure doses in various age groups of the study area.

Figure 4 presents a comprehensive overview of the yearly effective doses of ^222^Rn in water across different age brackets, encompassing infants, adults, and children. This summary encompasses both the inhalation and ingestion routes of exposure. According to the recommendations from reputable sources such as the World Health Organization (WHO) and the European Union (EU), the suggested annual permissible limit for radiation dose resulting from the consumption of drinking water stands at 0.1 mSv a^−1^ ^56^.

The annual mean effective doses of ^222^Rn due to inhalation and ingestion were calculated for each individual including different age groups: infants, children, and adults, and summarized (Table 2, Fig. 4). Results revealed the highest mean annual exposure doses for infants, child, and adults are (0.048, 0.036 and 0.035 mSv a^−1^) respectively (Table 2). The results revealed that annual effective doses of all age groups are below 0.1 mSv a^−1^ by the World Health Organisation (WHO). The mean annual effective doses of the study area were found within the WHO threshold (Fig. 4). The higher mean annual effective doses could cause various kinds of cancer in humans.

**Table 2:** Showing exposure doses in different age groups in water samples and average values.

Sam no.	Infant	Child	Adult	Sam no	Infant	Child	Adult
1	0.041	0.031	0.030	16	0.041	0.031	0.030
2	0.034	0.026	0.025	17	0.041	0.031	0.030
3	0.057	0.043	0.042	18	0.053	0.040	0.039
4	0.057	0.043	0.042	19	0.021	0.016	0.016
5	0.045	0.034	0.033	20	0.011	0.008	0.008
6	0.041	0.031	0.030	21	0.024	0.018	0.017
7	0.061	0.047	0.045	22	0.049	0.037	0.036
8	0.031	0.024	0.023	23	0.032	0.025	0.024
9	0.033	0.025	0.024	24	0.038	0.029	0.028
10	0.049	0.037	0.036	25	0.053	0.040	0.039
11	0.036	0.027	0.026	26	0.061	0.047	0.045
12	0.069	0.053	0.051	27	0.073	0.056	0.054
13	0.065	0.050	0.048	28	0.057	0.043	0.042
14	0.089	0.068	0.066	29	0.077	0.059	0.057
15	0.061	0.047	0.045	30	0.093	0.071	0.069
Average	0.048	0.036	0.035				

If the radiation dose in water intended for drinking is equal to or below 0.1 mSv a^−1^, it is considered safe and does not require any further action. If the dose from drinking water exceeds the annual safe limit of 0.1 mSv a^−1^, remedial measures are necessary to reduce the risk. According to a recent study conducted in the Mulazai area of Peshawar city, the average radiation exposure for individuals from water sources used for drinking purposes was found to be 0.0025, 0.0307, and 0.0333 mSv a^−1^ through ingestion, inhalation, and combined ingestion and inhalation (whole-body) showing in Table 3. These values are exemplary as they are well below the safety threshold of 0.1 mSv a^−1^ established by the World Health Organization in 2004. Consequently, there are no potential health risks associated with the presence of ^222^Rn in the drinking water of the study area. UNSCEAR has provided valuable data regarding the typical concentration of ^222^Rn in drinking water. According to their research in 2000, the average dose of radon radiation received through ingestion is measured at 0.002 mSv per year, whereas inhalation accounts for 0.025 mSv per year^41^. These findings strongly suggest that inhaling radon is the primary source of exposure when consuming water. Furthermore, this study reveals that the mean annual effective doses for the stomach and lungs resulting from radon-contaminated water are higher than the respective mean annual effective doses of 0.002 and 0.025 mSv per year reported by UNSCEAR for the same organs. The analysis of drinking water samples in this study revealed a considerably higher concentration of ^222^Rn compared to the samples collected from Coonoor. In the water samples of our study, the average concentration of ^222^Rn was found to be 12.05 Bq L^−1^. In contrast, previous studies conducted in Coonoor and Varahi Command areas of India^57,58^, as well as Murree in Pakistan^59^, reported mean concentrations of 1.20, 2.07, and 4.38 Bq L^−1^, respectively. Table 4 presents a comparison of the annual effective doses from drinking water between our study area and other regions and countries as documented in previous research.

**Table 3:** Showing radiation exposure through different pathways of the body.

Sample no.	EW Ing	EW Inh	EW total	Sample no.	EW Ing	EW Inh	EW total
1	0.0021	0.0252	0.0273	16	0.0021	0.0252	0.0273
2	0.00176	0.02117	0.02293	17	0.0021	0.0252	0.0273
3	0.00294	0.03528	0.03822	18	0.0027	0.0328	0.0355
4	0.00294	0.0353	0.03822	19	0.0011	0.0133	0.0144
5	0.00231	0.02772	0.03003	20	0.0005	0.0066	0.0071
6	0.0021	0.0252	0.0273	21	0.0012	0.0147	0.0159
7	0.00315	0.0378	0.04095	22	0.0025	0.0302	0.0328
8	0.001609	0.01931	0.02092	23	0.0017	0.0200	0.0217
9	0.001714	0.02056	0.02228	24	0.0020	0.0238	0.0258
10	0.00252	0.03024	0.03276	25	0.0027	0.0328	0.0355
11	0.001846	0.02216	0.02400	26	0.0032	0.0378	0.0410
12	0.00357	0.04284	0.04641	27	0.0038	0.0454	0.0491
13	0.00336	0.04032	0.04368	28	0.0029	0.0353	0.0382
14	0.00462	0.05544	0.06006	29	0.0040	0.0479	0.0519
15	0.00315	0.0378	0.04095	30	0.0048	0.0580	0.0628
Average	0.00257	0.03084	0.3341				
WHO	0.1

**Table 4:** The annual effective doses from drinking water in the study area compared to those of other regions and countries in previous studies.

Location	Country	Annual effective dose (mSv a^−1^)			References
		Ingestion	Inhalation	Mean effective dose	
Gilgit	Pakistan			0.626	^9^
Penang	Malaysia			0.77 ± 0.19	^27^
Rajasthan	India			1.1	^12^
Bannu	Pakistan			0.001–3.79	^32^
Nigeria	Nigeria			0.092	^47^
Agbabu	Nigeria			0.08	^28^
Iraq	Iraq			0.0004	^48^
Mirpur	Pakistan	0.004 ± 0.0003	0.045 ± 0.004	0.049 ± 0.01	^49^
Dhaka	Bangladesh	0.03	–	–	^50^
Kirkuk	Iraq	1.51 ± 1.22	10.1 ± 3.18		^52^
Rajasthan	India	0.001	0.0111	0.012	^51^
Karak	Pakistan	0.002 ± 0.0001	0.024 ± 0.001	0.026 ± 0.001	^53^
Peshawar	Pakistan	0.002 ± 0.0002	0.022 ± 0.002	0.024 ± 0.002	^43^
Mirpur	Pakistan	0.001	0.03 ± 0.01	0.03 ± 0.01	^31^

The Varahi and Coonoor regions in India were associated with annual whole-body doses of 0.0076 and 0.0102 mSv a^−1^, respectively. In contrast, the mean effective dose of ^222^Rn in the water samples of our study area was 0.0333 mSv a^−1^, which surpasses that of the Coonoor and Varahi regions. This discrepancy can be attributed to the geological characteristics of our study area and the elevated levels of ^222^Rn detected in its water sources. Similarly, the mean concentration of ^222^Rn (12.05 Bq L^−1^) and the total mean annual effective dose (0.0333 mSv a^−1^) in drinking water sources from our study area were higher than those observed in the University of Peshawar and its surrounding areas. In that particular region, the average ^222^Rn concentration was 8.80 Bq L^−1^, and the mean annual effective dose was 0.02403 mSv a^−1^ ^43^.

It is important to highlight that in our study area, more than 50% of the collected drinking water samples displayed levels of ^222^Rn that exceeded the recommended Maximum Contaminant Level (MCL) of 11.1 Bq L^−1^ for community water supplies, as specified by the US Environmental Protection Agency^45^.

The Mulazai region potentially contains concealed faults beneath or nearby; however, due to complete alluvial coverage and the absence of underground geological investigations, it is challenging to confirm this. If a fault does exist, the emission of ^222^Rn gas in the subsurface could travel upward through the porous pathways within the fault. This may pose a risk to humans utilizing groundwater for residential purposes, potentially leading to the development of stomach and lung cancer.

The heightened measurements could be attributed to a favorable connection between the levels of ^222^Rn and the depth in the vicinity, which grows more pronounced with increasing depth. Based on the geological examination, it is determined that the water sources in the research area originate from an aquifer consisting of Pleistocene-era deposits of sand, gravel, and boulders.

The Peshawar Basin receives sediments from both the southern Lesser Himalayas and the higher Himalayas in the north^36^. These sediments contain ^222^Rn, but their porous nature and diverse lithology make them prone to absorbing ^222^Rn through water infiltration, making them unlikely to reach underground water sources. By using deeper tube wells, there is a higher chance of encountering a thicker aquifer layer consisting of Pleistocene sand, gravel, and boulders, leading to an expected increase in ^222^Rn levels with greater depth. The uranium-rich sandy gravels at deeper levels are believed to originate from the northern higher Himalayas and are expected to have higher uranium contents compared to the uranium-poor sands and clays found at shallower depths in the boreholes. This aligns with findings from the Baoji region in China, which demonstrated a correlation between well-depth and ^222^Rn concentration in drinking water samples^60^. Consequently, precautionary measures should be taken, such as boiling water in well-ventilated areas, to minimize exposure to ^222^Rn in drinking water.

### Excess lifetime cancer risk

**Figure 5:**
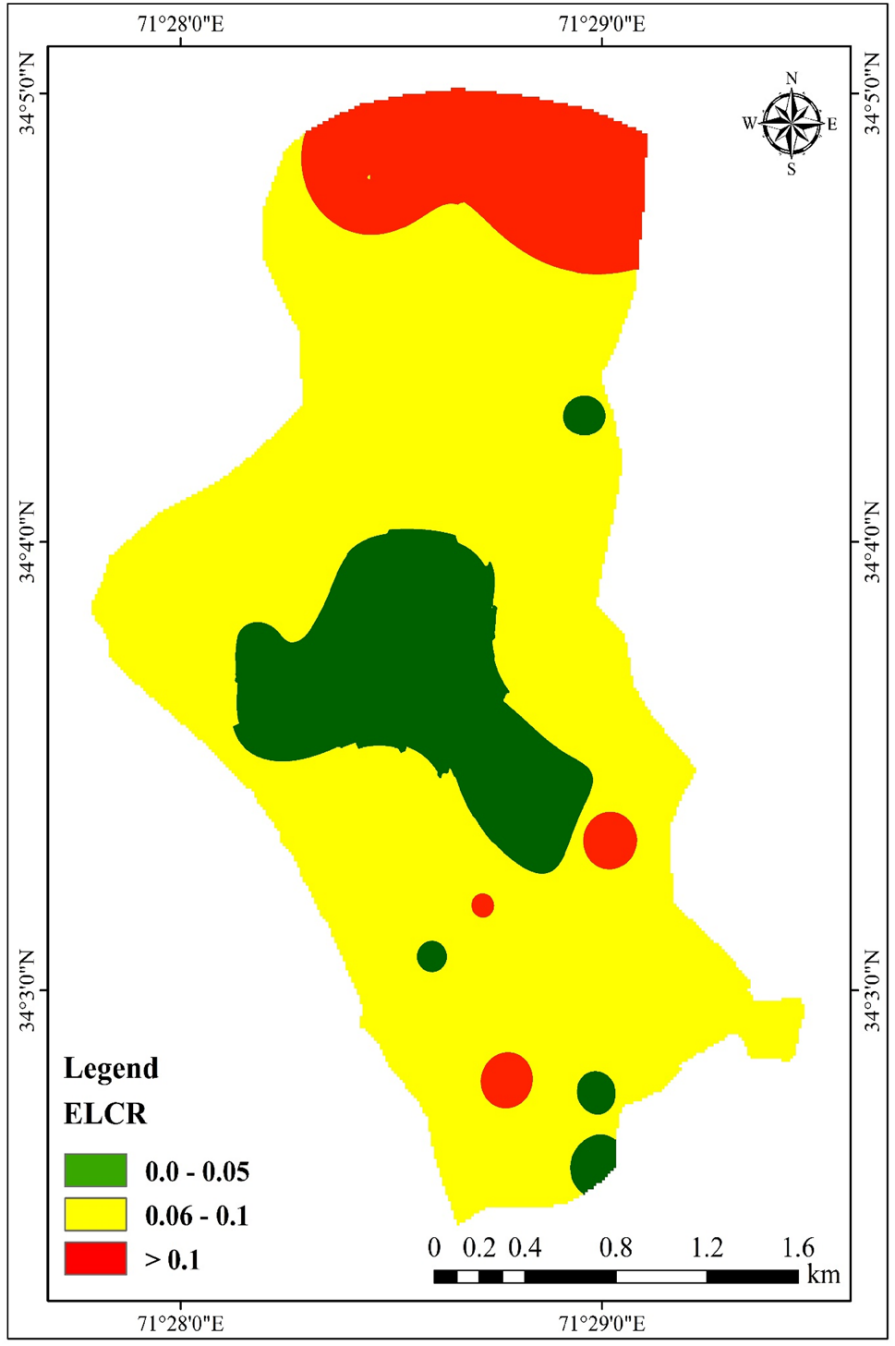
Geospatial map of Cancer risk in the water of the study area.

Excess vulnerability to cancer refers to the likelihood of developing cancer that surpasses the typical risk level of 240 due to prolonged exposure to carcinogenic substances. Previous studies by Freni ^61^ and Ibikunle et al. ^62^ have established this concept. In the current study, the average Excess Lifetime Cancer Risk (ELCR) value was determined to be 0.084 × 10^−3^ (Fig. 5). These findings were compared against the safety limit set by the US EPA (0.1 × 10^−3^) and were mostly found to be within an acceptable range, except for 30% of the groundwater samples collected in the area. This indicates a potential cancer risk for individuals exposed to the affected water in the future. The estimated ELCR in this study was higher than the reported ELCR in Vietnam^63^ but lower than the ELCR associated with exposure in Iran^64^.

## Conclusions

This study delves into the alarming presence of ^222^Rn in the drinking water of Mulazai, revealing a significant 54% of the tested samples had concentrations of ^222^Rn surpassing the EPA's suggested limit of 11.1 Bq L^−1^. This heightened level poses potential health risks due to prolonged exposure to radon, a known carcinogen. The estimated annual effective doses resulting from this exposure surpass the global average but remain under the safety limits set by the World Health Organization (WHO) and the European Union (EU). However, specific age groups, particularly infants, face a higher annual effective dose, calling for heightened concern and vigilance regarding their vulnerability to increased radon exposure. Despite these elevated concentrations, the measured levels fall within permissible limits outlined by international regulatory bodies, suggesting a level of safety in the immediate context.

The study underscores the critical need for sustained monitoring and proactive strategies to mitigate potential long-term health consequences stemming from elevated radon levels in drinking water. Collaborative efforts among policymakers, public health authorities, and local communities are imperative to ensure ongoing surveillance, effective mitigation measures, and public awareness campaigns.

## Data Availability

Upon request to the corresponding authors, data will be made available.
